# Multifactorial structure of cognitive assessment tests in the UK Biobank: A combined exploratory factor and structural equation modeling analyses

**DOI:** 10.3389/fpsyg.2023.1054707

**Published:** 2023-01-26

**Authors:** Liliana G. Ciobanu, Lazar Stankov, Muktar Ahmed, Andrew Heathcote, Scott Richard Clark, Eugene Aidman

**Affiliations:** ^1^Discipline of Psychiatry, The University of Adelaide, Adelaide, SA, Australia; ^2^School of Psychology, The University of Sydney, Sydney, NSW, Australia; ^3^School of Psychology, University of Newcastle, Sydney, NSW, Australia; ^4^School of Biomedical Sciences and Pharmacy, University of Newcastle, Callaghan, NSW, Australia; ^5^Decision Sciences Division, Defense Science and Technology Group, Adelaide, SA, Australia

**Keywords:** cognition, UK biobank, exploratory factor analysis, structural equation modeling, cognitive tests

## Abstract

**Introduction:**

The UK Biobank cognitive assessment data has been a significant resource for researchers looking to investigate predictors and modifiers of cognitive abilities and associated health outcomes in the general population. Given the diverse nature of this data, researchers use different approaches – from the use of a single test to composing the general intelligence score, *g*, across the tests. We argue that both approaches are suboptimal - one being too specific and the other one too general – and suggest a novel multifactorial solution to represent cognitive abilities.

**Methods:**

Using a combined Exploratory Factor (EFA) and Exploratory Structural Equation Modeling Analyses (ESEM) we developed a three-factor model to characterize an underlying structure of nine cognitive tests selected from the UK Biobank using a Cattell-Horn-Carroll framework. We first estimated a series of probable factor solutions using the maximum likelihood method of extraction. The best solution for the EFA-defined factor structure was then tested using the ESEM approach with the aim of confirming or disconfirming the decisions made.

**Results:**

We determined that a three-factor model fits the UK Biobank cognitive assessment data best. Two of the three factors can be assigned to *fluid reasoning (Gf)* with a clear distinction between *visuospatial reasoning* and *verbal-analytical reasoning*. The third factor was identified as a *processing speed (Gs)* factor.

**Discussion:**

This study characterizes cognitive assessment data in the UK Biobank and delivers an alternative view on its underlying structure, suggesting that the three factor model provides a more granular solution than *g* that can further be applied to study different facets of cognitive functioning in relation to health outcomes and to further progress examination of its biological underpinnings.

## 1. Introduction

The UK Biobank is a large-scale biomedical database and research resource, containing extensive genotyping and phenotypic information from half a million UK participants.^1^ It is a major contributor to the advancement of modern medicine and treatment and has enabled several scientific discoveries that improve human health (Sudlow et al., 2015). Cognitive assessment data in the UK Biobank has been a significant resource for researchers looking to investigate predictors and modifiers of cognitive abilities and associated health outcomes in the general population. While being one of the largest data sources available, its cognitive assessment component is not without limitations: it is brief and bespoke (non-standard) and is administered without supervision on a touch screen computer. Furthermore, not all participants completed the same number of tests and those who completed the same number of tests did not necessarily complete the same combination of tests. However, despite these challenges, some of the tests used have substantial concurrent validity and test–retest reliability (Fawns-Ritchie and Deary, 2020), yet with varying levels of stability of the scores over time (Lyall et al., 2016). While this evidence suggests acceptable psychometric properties of the UK Biobank cognitive assessment, spareness remains a concern. To maximize the sample size and, therefore, increase the statistical power of studies using the UK Biobank data, researchers employ different strategies. Some have used a single test, such as a Verbal Numerical Reasoning (Fluid Intelligence, FI) score or a reaction time (RT) score (Davies et al., 2016; Sniekers et al., 2017; Kievit et al., 2018; Lee et al., 2018; Savage et al., 2018). Others have extracted a ‘g-factor’ of general cognitive ability by aggregating several variables using Principal Component Analysis (PCA) or Confirmatory Factor Analysis (CFA; Lyall et al., 2016; Navrady et al., 2017; Cox et al., 2019; de la Fuente et al., 2021; Hepsomali and Groeger, 2021). However, this inconsistency in the definition of cognitive domains across different studies is a potential threat to replicability of the findings. Furthermore, most studies have used test scores that were neither adjusted for age nor standardized relative to a representative sample of the general population, despite the acknowledged lack of representativeness of the UK Biobank sample (Fry et al., 2017).

In an attempt to harmonize future studies of cognitive functioning using the UK Biobank data, Williams et al. (2022) developed a standardized measure of general intelligence, *g*, for most UK Biobank participants. While this is important for some applications, particularly for using general intelligence as a covariate, this measure does not capture adequately the multitude of cognitive test data in the UK Biobank.

We argue that neither a single test nor an aggregated *g* score – optimally capture the richness of cognitive testing data in the UK Biobank. The use of a single test is often too specific to be generalized to broader cognitive abilities, while *g* is too general to be used in practice where more targeted assessment of cognitive abilities is required. We suggest that an alternative multifactorial model of cognitive abilities developed through factor analysis and structural equation modeling, and use of these latent variables as an outcome measure is a better approach to capture the multitude of cognitive abilities in the UK Biobank.

We used framework provided by the Cattell-Horn-Carroll (CHC) theory of intelligence (Schneider and McGrew, 2018) to select nine UK Biobank cognitive measures for inclusion in the analyses. This allowed us to exclude some of the cognitive tests that have poor psychometric properties, too small N, and are used to assess specific clinical symptoms. Williams et al. (2022) provide detailed account of the reasons for excluding some cognitive measures, and for including eight of our selected tests in studies of cognitive abilities based on UK Biobank data. Second, it allowed us to classify the chosen cognitive variables into the broad dimensions/factors of the CHC theory – fluid intelligence (Gf), short-term working memory (Gwm), and processing speed (Gs).^2^ We hypothesized that these three factors may be able to be extracted from the UK Biobank cognitive testing data. In the next section we list the nine chosen tests and indicate what broad CHC factors we presumed they contribute to.

## 2. Methods

### 2.1. Study design and participants

UK Biobank (UKB) is a large prospective cohort of more than half a million participants aged 37–73 years, during recruitment between 2006 and 2010. Participants were recruited from a range of backgrounds and demographics and attended one of the 22 assessment centers where they completed baseline touchscreen questionnaires on sociodemographic factors (age, gender, ethnicity, and postcode of residence), behavior, and lifestyle (including smoking behavior and alcohol consumption), mental health, and cognitive function tests. The UK Biobank study was approved by the National Information Governance Board for Health and Social Care and Northwest Multicentre Research Ethics Committee (11/NW/0382). Participants provide electronic consent to use their anonymized data and samples for health-related research, to be re-contacted for further sub-studies, and for the UK Biobank to access their health-related records (Sudlow et al., 2015). This research has been conducted using the UK Biobank Resource under Application Number 71131.

### 2.2. Cognitive function assessments

At baseline, several cognitive tests were included in the UK Biobank, all of which were administered *via* a computerized touchscreen interface. In addition to the data collected from assessment center visits, the UK Biobank collected enhancement data using web-based questionnaires. The baseline cognitive function tests along with two additional tests were administered as an online questionnaire. A sub-sample of around 20,000 participants subsequently underwent a repeat assessment. During the repeated assessment, all participants completed physical, medical, sociodemographic, and cognitive assessments. Some cognitive tests were added/removed at different stages of baseline assessment, the number of participants with complete data varies across tests (Fawns-Ritchie and Deary, 2020). Cognitive tests included in the current analyses and CHC factors they were hypothesized to measure:

- Fluid intelligence (Gf): (1) Matrix Pattern Recognition (MPR); (2) Tower Rearrangement (TR); (3) Fluid Intelligence (FI); also labeled *verbal-numerical reasoning* by Lyall et al. (2016); (4) Paired-associate learning (PAL).
- Short-term working memory (Gwm): (5) Numeric Memory (NM); (6) Symbol-digit Substitution (SDS); (7) Pairs Matching (PM).
- Processing speed (Gs): (8) Reaction Time (RT); (9) Trail Making (TM).

A list and description of each cognitive measure used in the UK Biobank is available at https://biobank.ctsu.ox.ac.uk/crystal/label.cgi?id=100026.

To characterize the population with different cognitive tests an approach should be applied that allows for the analysis of data containing several groups of variables of different nature compiled within the same group of observations (individuals; Izquierdo et al., 2014). To this end, we applied an Exploratory Factor Analysis (EFA), and Exploratory Structural Equation Modeling (ESEM) on 3,425 study participants with completed data on nine cognitive tests at baseline and repeated assessments after excluding participants who were diagnosed with psychiatric and neurological disorders. The flow diagram for the cohort definition and the list of diseases excluded from the current analysis is in Supplementary Figure S1.

### 2.3. Statistical analysis

The baseline characteristics of participants are presented as means (SD) or median (interquartile range) for continuous variables and frequency (percentage) for categorical variables. The Kaiser-Meyer-Olkin (KMO) statistic for factor adequacy (Kaiser, 1974) and Bartlett’s test of sphericity (Bartlett, 1954) were applied to test the factorability of the data. After confirming that the correlation matrix was factorable, we carried out a series of exploratory factor analyses (EFA) using the maximum likelihood (ML) method of extraction followed by the “Promax*”* method of oblique rotation. Oblique factor rotations are commonly used in psychometric studies since they provide simple structure solutions that are easier to interpret than unrotated principal components (or factors). Promax rotation is a widely accepted efficient method of rotation (Finch, 2006). The results from the alternative “oblimin” rotation are included in the Supplementary Tables S9–S11 (Supplementary material, p. 18–20).

To determine the appropriate number of factors to retain, we analyzed the correlation matrix to examine the scree plot of the successive eigenvalues. Eigenvalues are a measure of the amount of variance accounted for by a factor, and so they can be useful in determining the number of factors that we need to extract. We generated a scree plot of eigenvalues for all factors and then looked to see where they drop off sharply. We also ran a “Parallel” analysis by comparing the solution (observed eigenvalues of a correlation matrix) with those from a random data matrix of the same size as the original (Horn, 1965). The number of simulated analyses [number of iterations] to perform in the parallel analysis was set to 10,000 against the default value of 20. The three-factor EFA solution was examined further.

The dataset was then analyzed using the exploratory structural equation modeling (ESEM) approach with the aim of confirming or disconfirming the decisions made with EFA (Beran and Violato, 2010). This analysis requires identifying the model, collecting, and screening data appropriate for the analyses, estimating the parameters of the model, assessing the fit of the model to the data, interpreting the model’s parameters, and evaluating the plausibility of competing models. We set the model by identifying anchor variables from EFA as an *a priori* hypothesis. We also performed several additional sensitivity analyses. Further details of these analyses can be found in the Supplementary material. All analyses were conducted using R Version 4.0.5 (R Foundation for Statistical Computing) using *psych* and *lavaan* packages.

## 3. Results

### 3.1. Baseline characteristics

Data from a total of 3,425 participants were used in the current analysis. Of the 3,425 participants, almost 49.8% of the overall sample were women, and the mean ± SD age of participants was 55 ± 7.5 years (range 37–73 years). Nearly 44.6% of them had completed a college education. The highest proportion of participants (97.8%) were of white ethnic background (more details in Supplementary Table S1). The schematic presentation of how study participants are enrolled in the UKB and included in this analysis is shown in Supplementary Figure S1.

### 3.2. Descriptive statistics

Table 1 presents means, standard deviations and correlations between nine cognitive variables. The highest means are on two measures – Reaction Time and Trails Making - that are scored in terms of time needed to carry out the task. For these tests, shorter time indicates better performance. This is the reason for the presence of negative correlations they have with accuracy scores of most other variables in the battery. Another test that has negative correlations is Pairs Matching which, although capturing an aspect of speed due to its time limit is primarily characterized by the nature of scoring. Its total score is the Number of incorrect matches in a given round. Although it is typically claimed that cognitive tests tend to show positive correlations, it is apparent that the nature of scoring affects the sign of their correlations.

**Table 1:** Descriptive statistics for nine cognitive function tests in the UK Biobank.

No	Cognitive function tests	Mean	SD	Symbol digit substitution	Fluid intelligence score	Trail Making	Numeric memory	Pairs matching	Reaction time	Matrix pattern completion	Tower rearrangement	Paired associate learning
1	Symbol digit substitution	18.87	5.22	1.00								
2	Fluid intelligence score	6.69	2.00	0.25	1.00							
3	Trail Making	548.45	268.33	−0.31	−0.28	1.00						
4	Numeric memory	6.92	1.36	0.15	0.35	−0.22	1.00					
5	Pairs matching	3.62	2.95	−0.19	−0.13	0.14	−0.11	1.00				
6	Reaction time	543.42	111.67	−0.24	−0.10	0.13	−0.05	0.13	1.00			
7	Matrix pattern completion	7.98	2.13	0.36	0.38	−0.28	0.23	−0.19	−0.17	1.00		
8	Tower rearrangement	9.92	3.24	0.35	0.31	−0.25	0.21	−0.20	−0.20	0.35	1.00	
9	Paired associate learning	7.00	2.46	0.25	0.31	−0.19	0.21	−0.17	−0.11	0.28	0.25	1.00

It is also necessary to point out to the size of correlation coefficients in Table 1 – their (absolute) values vary from close to zero to 0.38 and the average is around 0.20s. This means that the tests have less in common than the average correlation of 0.29 reported by Carroll (2009) after re-analyzing a large number of studies of intelligence. Lower average correlation leads to a reduced strength of the general factor (Stankov, 2002). However, Bartlett’s test of sphericity produced a statistically significant value (*χ*^2^ = 4181.22, *p*-value <0.00, df = 36), implying that factor analysis may be carried out with our data. This test evaluates whether the variables intercorrelate at all, by comparing the observed correlation matrix with an “identity matrix” (a matrix in which all diagonal elements are 1 and all off-diagonal elements are 0). The overall KMO statistic was 0.84, also implying that factor analysis can be carried out.

### 3.3. Exploratory factor analysis

**Figure 1:**
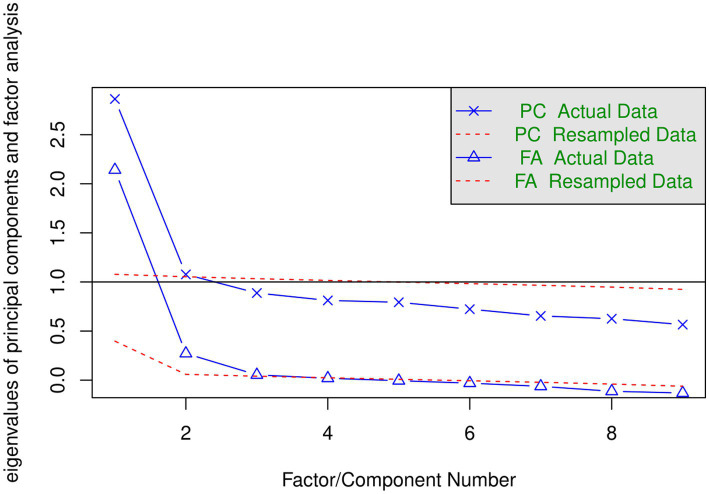
Scree plots for PCA and FA runs.

We employed several criteria to decide how many factors to extract. Figure 1 presents the “scree plots” for two sets of 9 eigenvalues. The solid blue line in the top part is based on the extraction of principal components (PCA) and solid line in the bottom part is based on factor analysis (FA, diagonal values in the matrix of correlations are replaced by the communalities). One criterion is the number of eigenvalues form PCA that are greater than 1 (horizontal line) in the top panel. This suggests the extraction of two factors. Another criterion is based on parallel analysis. Dotted red lines in Figure 1 represent the plot of the eigenvalues based on simulated data. The number of factors to be extracted is indicated by the crossover of the two (solid and dotted) lines. Each point on the solid blue line that lies above the corresponding simulated data line is a factor or component to extract. According to this criterion, 2 components in the PCA analysis lie above the corresponding simulated data line and 3 factors in the FA analysis lie above the corresponding simulated data line.

We ran three EFA analyses that extracted 1, 2, and 3 factors using the maximum likelihood procedure. This method of extraction provides a test of the hypothesis that the obtained factors are sufficient. For the two-factors solution the chi-square statistic is 38.37 (df = 19) and the value of *p* is borderline 0.005. For the three-factors solution presented in Table 2 chi-square statistics is 15.14 (df = 12) and the value of *p* is acceptable 0.234. Therefore, three factors are indicated by both the scree plot based on FA and by the maximum likelihood’s chi-square statistics. The outcomes of the one-and three-factors solutions will be discussed below. Most of the relevant aspects of the two-factors solution are captured by the three-factors solution.

**Table 2:** One-factor and three-factors solutions for cognitive tests in the UK Biobank.

Cognitive tests	One-factor solution			Three-factors solution			
	Factor 1	Uniqueness		Factor 1	Factor 2	Factor 3	Uniqueness
Symbol digit substitution	0.558	0.689		0.663			0.598
Fluid intelligence	0.572	0.673			0.746		0.476
Trail making	−0.474	0.776				0.973	0.005
Numeric memory	0.406	0.835			0.492		0.770
Pairs matching	−0.314	0.901		−0.343			0.887
Reaction time	−0.285	0.919		−0.469			0.854
Matrix pattern completion	0.622	0.613		0.412	0.265		0.623
Tower rearranging	0.574	0.671		0.500			0.657
Paired associate learning	0.467	0.782		0.229	0.308		0.776
							
Proportion of variance	0.238			0.140	0.113	0.106	
Cumulative variance	0.238			0.140	0.252	0.358	
							
Factor Correlations:	Factor 1			Factor 1	Factor 2	Factor 3	
Factor 1	1.00		Factor 1	1.00			
			Factor 2	−0.34	1.00		
			Factor 3	0.20	0.20	1.00	

### 3.4. Exploratory factor analysis: One factor solution

The left-hand side of Table 2 presents the 1-factor solution. We feel that it is important to present this solution since much of the published work based on UK Biobank has been focused on the general intelligence or *g-*factor that is often understood as the first factor from a battery of cognitive tests.

Two points are worth noting. First, the proportion of variance accounted for by the first factor is smaller than typically found with tests of intelligence. Stankov (2002) reported that proportion of the total variance captured by the first principal component in Carroll (2009) analyses is about 0.350. The first eigenvalue for the principal component solution in the present study with 9 variables is 2.863, indicating that the proportion of total variance accounted for by the first component is 0.318. As can be seen in Table 2, the first FA factor accounts for 0.238 proportion of total variance.

Second, the columns in Table 2 present tests’ loadings (i.e., variables’ weights) on a given factor. The columns labeled “Uniqueness” shows that much of the variance is unaccounted for by the factor(s). It is noticeable that elements in both columns vary in size and there are even negative loadings of the three variables that have negative raw correlations in Table 1. Particularly high are the uniqueness’ of the Pairs Matching (0.901) and Reaction Time (0.919). These tests are poor measures of the general factor in the present study, and it can be argued that processing speed is not an important aspect of intelligence in the UK Biobank dataset.

Both findings – low proportion of variance and low communality of the processing speed measures – suggest that it would be useful to focus on additional factor(s).

### 3.5. Exploratory factor analysis: Three factors solution

The factor pattern matrix for the three-factors solution is shown on the right-hand side of Table 2. It is based on maximum likelihood extraction and Promax rotation. Factor loadings higher than 0.20 for a given factor, uniqueness’, and correlations between the factors are displayed. Together, three factors capture 0.358 proportion of the total variance. For this solution, the Chi-square goodness-of-fit statistics = 15.14, df = 12 (*p* = 0.234) indicates that we fitted an appropriate model to capture the full dimensionality of the data.

Factor 1 is defined by six variables, with Symbol Digit Substitution having the highest (0.663) and Paired Associate Learning having the lowest (0.229) loadings. Factor 2 has the highest loading on Fluid Intelligence (0.746) and the lowest on Matrix Pattern Completion (0.265). Importantly, Factor 3 is a singlet, having a noteworthy loading from the Trail Making (0.973) test only. Substantive interpretations of the factors will be provided in a latter section of this paper. It can be noted, however, that two tests that were hypothesized to define short-term working memory (Gwm) in CHC theory - Numeric Memory and Symbol Digit Substitution-load on different factors and therefore the existence of Gwm is not supported by the EFA analyses.

However, it is necessary to make further comments about the nature of the Trail Making test. Our hypothesis was that this test, together with Reaction Time and Pairs Matching will define the processing speed (Gs) broad factor from the CHC theory of intelligence. Thus, although our expectation was that all three will define the same factor, Reaction Time and Pairs Matching retained their loadings on Factor 1. This outcome led us to consider postulating that these three tests may load on the same factor by using confirmatory approach as the next step in the analysis.

Promax is the oblique rotation, and therefore it is expected that factors will be correlated. As can be seen in the factor correlation matrix at the bottom part of Table 2, the correlations are small to moderate in size. Factors 1 and 2 have negative correlation (−0.34), reflecting again reverse scoring of two tests that load on Factor 1 and factor 3 has the same size correlation (0.20) with both Factors 1 and 2. It is highly unlikely that this pattern of correlations would lead to the emergence of a strong second-order factor.

### 3.6. Exploratory structural equation modeling: Modifying the EFA solution

Statistical procedures of ESEM were developed by Asparouhov and Muthén (2009). As pointed out by Marsh et al. (2014) one of its applications is in the area usually addressed by the confirmatory factor analysis (CFA) – i.e., testing if a particular structural model holds within a given dataset. We report the outcomes of two ESEM analyses carried out with the UK Biobank data.

First, we test the model based on the three-factors EFA solution presented in the right-hand side of Table 2. The input (i.e., anchors) were factor coefficients (loadings) for each factor from that solution. ESEM program uses maximum likelihood method in an iterative way to estimate the extent to which the model predicts the values of the sample covariance/correlation matrix.

The first line in Table 3 presents goodness-of-fit indices for this model. All these indices indicate poor fit. Thus, significant Chi-square test (*p*-value <0.001), suggests that the model is too simple to properly represent the data structure. Also, the acceptable RMSEA and SRMR values should be lower than 0.06, and CFI and TLI need to be above 0.90. This suggests that the model can be improved if modifications are made by introducing additional path coefficients or covariances (Kang and Ahn, 2021).

**Table 3:** Goodness-of-fit indices for three-factor solutions in Tables 2, 4.

Three-factors solution	*χ* ^2^	df	BIC	RMSEA	SRMR	CFI	TLI
EFA-based model (right-hand side of Table 2)	1499.3	33	84951.72	0.11	0.13	0.65	0.62
Modified solution (Table 4)	389.08	33	83841.43	0.056	0.054	0.914	0.906

*χ*^2^, chi-square statistic; df, degree of freedom; RMSEA, root mean squared error of approximation; SRMR, standardized root mean square residual; CFI, comparative fit index; TLI, Tucker-Lewis index; BIC, Bayesian information criteria (Dai et al., 2020).

Second, we tested a modified model in the next run. The following modifications were introduced: (a) Pairs Matching, and Reaction Time tests were removed from Factor 1 and were given new loadings (0.300 each) on Factor 3; (b) The loading of the Trail Making test on factor 3 was set to 0.700; (c) The loading of the Paired Associate learning test on Factor 1 was removed; and (d) The loading of Matrix Pattern Completion on Factor 2 was set to 0.300. All input data for this modified run are presented in the left-hand side “Estimates” section of Table 4.

**Table 4:** A three-factors model of cognitive tests based on ESEM analysis.

Cognitive tests	Estimates			Completely standardized solution		
	Factor 1	Factor 2	Factor 3	Factor 1	Factor 2	Factor 3
Symbol digit substitution	0.663			0.651		
Fluid intelligence		0.746			0.734	
Trail making			0.700			0.660
Numeric memory		0.492			0.488	
Pairs matching			0.300			0.301
Reaction time			0.300			0.300
Matrix pattern completion	0.412	0.300		0.404	0.294	
Tower rearranging	0.500			0.514		
Paired associate learning		0.308			0.319	
				Correlations		
			Factor 1	1.000		
			Factor 2	0.660	1.000	
			Factor 3	−0.855	−0.645	1.000

**Figure 2:**
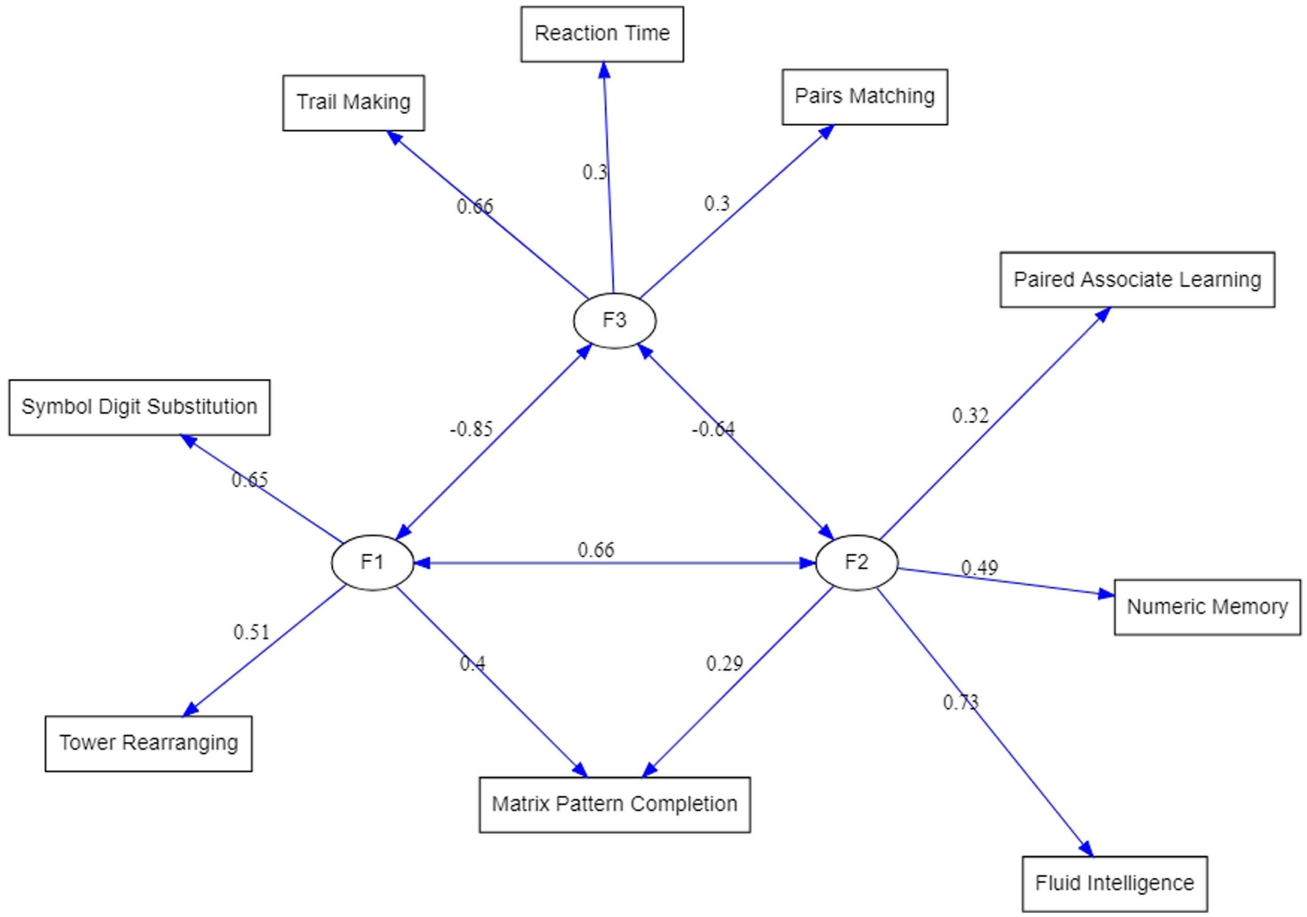
ESEM-reconstructed factorial structure of the cognitive test variables from the UK Biobank. Circles represent factors. The double-sided arrows indicate covariances.

Testing the model with the proposed modifications resulted in a greatly improved fit indices listed in the second row of Table 3. In comparison to the EFA-based model, Chi-square has been reduced although still significant, but all other goodness-of-fit indices have reached acceptable levels. Therefore, the structure displayed on the right-hand side of Table 4 under the heading “Completely standardized solution” represents the three latent dimensions underlying cognitive tests in the UK Biobank dataset. Figure 2 is a graphical display of this final model.

Correlations among the factors are also shown in the lower part of Table 4. These can be compared with the three-factors EFA solution in Table 2. Two features stand out. First, factor intercorrelations produced by the ESEM are much higher than those from EFA and it can be expected that they will lead to an identification of the second-order factor, which corresponds to *fluid reasoning, Gf*. Second, correlation between Factor 1 and Factor 2 was negative in the EFA solution but moving Pairs Matching and Reaction Time tests to load on Factor 3 led to its negative correlation with the other two factors. While higher correlations may be important for the interpretation of the results, changes in negative correlations are simply a consequence of reverse scoring of the tests that load on Factor 3.

## 4. Discussion

Being a valuable source of a population level cognitive functioning data for research purposes, the bespoke format of the UK Biobank cognitive assessment provides challenges to realizing its scientific potential. While using a single test or an extraction of the general intelligence score, *g*, are common ways of dealing with the data limitations, these approaches themselves are limiting. We propose an alternative multifactorial approach that uses a CHC framework to analyze selected tests to capture different facets of the UK Biobank cognitive assessment without being too specific, as in a case with one single test, or too general, as in a case of using *g*.

### 4.1. Factor interpretation

Using factor analysis and structural equation modeling we show that the three-factor model that is based on nine cognitive tests available in the UK Biobank provides a more refined solution for capturing various facets of cognitive abilities. Due to the lack of appropriate tests in the UK Biobank, all three factors are of a fluid intelligence type that was initially defined by Cattell (1941) and further elaborated by Horn and Cattell (1966) and Reynolds et al. (2022). In our model, two out of the three factors can be assigned to *fluid reasoning (Gf)* described in Cattell–Horn–Carroll (CHC) theory as a broad ability to reason, form concepts, and solve problems using unfamiliar information or novel procedures, and one factor - to *processing speed (Gs)* - the ability to perform automatic cognitive tasks, particularly when measured under pressure to maintain focused attention (Flanagan et al., 2007).

The following discussion is primarily building upon the three-factor structural equation model as the most mature model of the UK Biobank cognitive assessment developed in this study (Figure 2).

The first factor (F1) was defined by Symbol Digit Substitution (SDS, 0.65 F1 loading), Tower Rearranging (TR, 0.51 F1 loading) and Matrix Pattern Completion (MPC, 0.4 F1 loading) tests. These tasks are well known measures of a fluid reasoning, however, given a dominating role of a visuospatial processing in these tests, F1 can be seen as a *visuospatial Gf* factor. The second factor (F2), comprising of Fluid Intelligence or *‘verbal-numerical reasoning’* (FI, 0.73 F2 loading), Numeric Memory (NM, 0.49 F2 loading), Paired Associate Learning (PAL, 0.32 F2 loading) and Matrix Pattern Completion (MPC, 0.29 F2 loading) tests (Figure 2). While F2 tests, like the F1 tests, are known to measure broad *Gf* ability, FI, NM, and PAL are based on verbal-auditory processing, thus, F2 can be viewed as *verbal-analytic Gf* factor. An interesting question arises about the MPC test that is shared between F1 (0.4 loading) and F2 (0.29 loading): Why being a classical visuospatial test [MPC is a part of the Wechsler Adult Intelligence Scale (WAIS)] does it also appear in a so-called *verbal-analytic Gf* factor? The answer may lay in the relationship between language and thought, which is an intriguing and challenging area of inquiry for scientists across many disciplines. In neuropsychology, researchers have investigated the inter-dependence of language and thought by testing individuals with compromised language abilities and observing whether performance in other cognitive domains is diminished. They found that individuals with severe comprehension deficits such as those with Wernicke’s aphasia appear to be especially impaired non-verbal reasoning tasks (Kertesz and McCabe, 1975; Hjelmquist, 1989; Baldo et al., 2005, 2015). Together, these findings suggest that language supports complex reasoning, possibly due to the facilitative role of verbal working memory and inner speech in higher mental processes.

The CHC theory distinguishes *auditory (Ga)* and *visual (Gv)* cognitive processing (Schneider and McGrew, 2018) as separate broad abilities, however, it appears that the UK Biobank cognitive assessment tasks measure these processes in conjunction with *reasoning*. The distinction of the two different types of reasoning in *Gf* observed in our model is also supported by converging evidence from imaging studies of brain functional connectivity. Thus, Jung and Haier (2007) have proposed the empirical-based parieto-frontal integration theory (P-FIT) of intelligence, which has been proposed as one the most promising theories to guide research on the biology underpinning human intelligence (Deary et al., 2010). The P-FIT states that large scale brain networks that connect brain regions, including regions within frontal, parietal, temporal, and cingulate cortices, underlie the biological basis of human intelligence. Several studies have provided further support for this theory, identifying grey matter correlates of fluid, crystallized, and spatial intelligence (Colom et al., 2009), separable networks for top-down attention to auditory non-spatial and visuospatial modalities (Braga et al., 2013), and separate but interacting neural networks in specific brain regions for *visuospatial* and *verbal-analytic visuospatial reasoning* (Chen et al., 2017). Together, these findings provide an empirical biology-based account for the first two factors in our model of the UK Biobank cognitive assessments.

The third factor (F3) was composed of Trail Making (TM, 0.6 F3 loading), Reaction Time (RT, 0.3 F3 loading) and Pairs Matching (PM, 0.3 F3 loading) tests (a single-test factor with a 0.97 TM loading in the exploratory factor analysis, Table 3). As can be seen (Figure 2), the largest loading on F3 was from the TM - a neuropsychological test of *visual attention* and *task switching* that can provide information about visual search speed, scanning, speed of processing, mental flexibility, as well as executive functioning (Arnett and Labovitz, 1995). Given that the TM has been shown to be both phenotypically and genetically strongly associated with processing speed (Edwards et al., 2017), it can be advised that F3 is predominantly a *processing speed (Gs)* factor. It is worth noting that all three tests are of a *visual processing* nature, and the correlation of F3 with the *visuospatial reasoning,* F1, factor (*r* = 0.85) is higher than with the *verbal-analytic*, F2, factor (*r* = 0.64), which suggests that visual perception, as an integral component of these tasks, plays an important role in this factor.

It is well established that slowed processing speed contributes to cognitive deficits in amnestic and non-amnestic mild cognitive impairment (Edwards et al., 2017; Daugherty et al., 2020). Tests measuring processing speed can be used as a cognitive marker in the differential diagnosis of mild neurocognitive disorders (NCD; Lu et al., 2017). As F3 is comprised of the three components – a basic measure of processing speed (RP test), visual memory (PM test) and a classic test for cognitive impairment (TM test; Cahn et al., 1995), it can be suggested that a UK Biobank latent variable of processing speed composed of TM, RT, and PM could be more sensitive in detecting cognitive impairment than each of the tests alone. However, more empirical evidence is required to support this observation.

### 4.2. Limitations

In this study, we used data from a total of 3,425 participants of white ethnicity – only those who had a complete set of cognitive assessment scores across all nine tests. While this has reduced our sample size considerably, it did not significantly affect the statistical power for our analyses; but to some extent limits the generalisability of the results. Our model structure may not hold true for a larger subset of the UK Biobank cognitive data, where there is systematic missing data from incomplete assessment, as well as missing random and not at random (nonignorable) data.

### 4.3. Conclusion and future directions

Cognitive assessment data in the UK Biobank has been a significant resource for researchers looking to investigate predictors and modifiers of cognitive abilities and associated health outcomes in the general population. However, these data are not without limitations. Extracting *g* from different cognitive tests is a common way to overcome these limitations. In this study, use a CHC framework to characterize the cognitive assessment data for nine cognitive tests in the UK Biobank and deliver an alternative view of its underlying structure, suggesting that the three-factor model provides a more granular solution than *g.* Using this multifactorial model in conjunction with genetic and brain imaging data from the UK biobank could provide novel insights into the biological mechanisms of *processing speed*, *visuospatial* and *verbal-analytic visuospatial reasoning*. These findings would add to the established genetic (Davies et al., 2018) and structural brain imaging (Cox et al., 2019) correlates of general intelligence, *g* already derived in the UK Biobank. The model can also be applied to study the relationships between risk factors, health outcomes and the specified cognitive dimensions to further progress in understanding of their biological underpinnings.

## Data availability statement

Publicly available datasets were analyzed in this study. This data can be found here: UK Biobank https://www.ukbiobank.ac.uk/.

## Ethics statement

The studies involving human participants were reviewed and approved by the National Information Governance Board for Health and Social Care and Northwest Multicentre Research Ethics Committee (11/NW/0382). The patients/participants provided their written informed consent to participate in this study.

## Author contributions

LC and LS designed the study and took the lead in writing the manuscript. MA carried out the analyses. SC, EA, LS, and AH contributed to the interpretation of the results. EA supervised the project. All authors provided critical feedback and helped shape the research, analysis, contributed to the article, and approved the submitted version.

## Funding

This work was supported by the Australian Army Headquarters (AHQ).

## Conflict of interest

The authors declare that the research was conducted in the absence of any commercial or financial relationships that could be construed as a potential conflict of interest.

## Publisher’s note

All claims expressed in this article are solely those of the authors and do not necessarily represent those of their affiliated organizations, or those of the publisher, the editors and the reviewers. Any product that may be evaluated in this article, or claim that may be made by its manufacturer, is not guaranteed or endorsed by the publisher.

## Footnotes

^1^ https://www.ukbiobank.ac.uk/

^2^Another important CHC factor, crystallized intelligence (Gc) which refers to the ability to comprehend and communicate culturally valued knowledge developed through learning and acculturation, is not sufficiently assessed by the available UK Biobank cognitive tests.
