# Supplementary material for: Multifactorial structure of cognitive assessment tests in the UK Biobank: A combined exploratory factor and structural equation modeling analyses
Source: Front Psychol. 2023 Jan 26;14:1054707. doi: 10.3389/fpsyg.2023.1054707 (PMC9937787; doi:10.3389/fpsyg.2023.1054707)
Supplement: Supplementary file 1 [file Table_1.DOCX]

Supplementary Materials

# Cohort characteristics

**Supplementary Table 1.** The baseline characteristics for the cognitive cohort in the UK Biobank

| Characteristics | Total, (n=3,425) n (%) |
| --- | --- |
| Gender |  |
| Male | 1,719 (50.19) |
| Female | 1,706 (49.81) |
| Age at first assessment |  |
| 39-49 years | 945 (27.59) |
| 50-59 years | 1,322 (38.60) |
| 60-73 years | 1,158 (33.81) |
| BMI (kg/m^2^) |  |
| Underweight[<18.5kg/m2] | 19 (0.55) |
| Normal [18.5-25kg/m2] | 1,273 (37.17) |
| Overweight[25-30kg/m2] | 1,515 (44.23) |
| Obese[≥30kg/m2] | 618 (18.04) |
| Smoking status |  |
| Never | 2,087 (60.93) |
| Previous | 1,134 (33.11) |
| Current | 198 (5.78) |
| Missing | 6 (0.18) |
| Frequency of alcohol drunk on a typical drinking day |  |
| Never | 1,295 (37.81) |
| 1 or 2 | 1,040 (30.36) |
| 3 or 4 | 580 (16.93) |
| 5 or 6 | 284 (8.29) |
| 7, 8 or 9 | 161 (4.70) |
| 10 or more | 61 (1.78) |
| Missing | 4 (0.12) |
| Educational status |  |
| None | 204 (5.96) |
| College or University degree | 1,526 (44.55) |
| A levels/AS levels or equivalent | 439 (12.82) |
| O levels/GCSEs or equivalent | 688 (20.09) |
| CSEs or equivalent | 178 (5.20) |
| NVQ or HND or HNC or equivalent | 203 (5.93) |
| Other professional qualifications | 175 (5.11) |
| Missing | 12 (0.35) |
| Ethnicity |  |
| White | 3,332 (97.28) |
| Mixed | 21 (0.61) |
| Asians | 50 (1.46) |
| Blacks | 15 (0.44) |
| Preferer not to answer | 7 (0.20) |

# Cognitive function measurements in the UK Biobank

For the exploratory factor analysis, we have used nine cognitive tests from the UKB datasets. The description of each test and how they are measured in the baseline and repeat assessment is summarized in this document.

During the main phase of recruitment, several cognitive function tests were performed in the following order:

• Prospective memory test – Part 1

• Pairs memory test

• Numeric memory test

• Fluid intelligence test

• Reaction time (Snap)

• Trail making test

• Symbol digit substitution test

• Picture vocabulary test

• Paired associate learning test – Part 1

• Matrix pattern completion

• Paired associate learning – Part 2

• Tower rearranging

• Prospective memory test – Part 2

Several of the cognitive function tests administered via touchscreen at the baseline Assessment Centre were re-implemented as web-based questionnaires, along with two additional cognitive function tests, and participants were invited to complete them remotely.

The order of the tests was

• Device and Mood

• Fluid intelligence

• Trail making

• Symbol digit substitution

• Pairs matching

• Numeric memory

Because it was possible to complete the modules over a number of sessions the Device/Mood questions were asked at the beginning of each online session. Hence some people will have multiple sets of answers for Device/Mood, and these can be related to their answer to the other modules by comparing their entry timestamps.

During the pilot phase of recruitment (2006), a reaction time test was also performed however the results were felt to be of insufficient quality to present due to inconsistencies in the timing routines of the various browsers and devices used by participants to perform the test. The data from the pilot and the main phase of recruitment are therefore presented separately.

***Fluid intelligence score (Field ID 20016) Category Fluid Intelligence***

This category contains data on questions designed to assess 'Fluid intelligence' (i.e., the capacity to solve problems that require logic and reasoning ability, independent of acquired knowledge). The participant has 2 minutes to complete as many questions as possible from the test.

In this analysis, we have used the fluid intelligence score. It is a simple unweighted sum of the number of correct answers given to the 13 fluid intelligence questions. Participants who did not answer all of the questions within the allotted 2-minute limit are scored as zero for each of the unattempt questions.

At the start of the test, the following text is shown:

“In this next test you will have a maximum of two minutes to answer as many questions as possible. Don’t spend too long on any one question and you can skip any question if you wish”

The choice of response is offered:

1. Begin check

2. I am unable to try this

If the participant is unable to try the test, this game is skipped, and they proceed to the Reaction time (Snap) touch-screen test.

If the participant selects 1, a timer is started and the questions are displayed in sequence, until 2 minutes have elapsed from the start of timing. Once the participant has completed the last question displayed, they proceed to the Reaction time (Snap) touch-screen test.

***Maximum digits remembered correctly (Field ID 4282) Category Numeric Memory***

In this analysis, we used the maximum digits remembered correctly from the numeric memory category and measured in the following procedure.

Longest number correctly recalled during the numeric memory test. A value of -1 is recorded if the participant chose to abandon the test before completing the first round.

This category contains data on a test designed to assess numeric short-term memory, as part of the touchscreen questionnaire. The participant was shown a 2-digit number to remember. The number then disappeared and after a short while they were asked to enter the number onto the screen. The number became one digit longer each time they remembered correctly (up to a maximum of 12 digits). Data collected include the number of digits and value of the number, the length of time the number was displayed, the time that the participant first entered and last entered a digit, the time taken to complete the test, the value of the number entered by the participant, whether or not the participant was correct, the maximum number of digits remembered, and whether the test was completed. This test is available for a subset of participants.

***Number of incorrect matches in round (Field ID 399) Category Pairs Matching***

This category contains data on 'pairs' matching tests. Participants are asked to memorize the position of as many matching pairs of cards as possible. The cards are then turned face down on the screen and the participant is asked to touch as many pairs as possible in the fewest tries. Multiple rounds were conducted. The first round used 3 pairs of cards and the second 6 pairs of cards.

A value of 0 indicates the participant made no mistakes. This variable has three data items at baseline, and we used the second data item recorded as 399_0_2

***Mean time to correctly identify matches (Field ID 20023) Category Reaction Time***

This category contains data on a test to assess reaction time and is based on 12 rounds of the card-game 'Snap'. The participant is shown two cards at a time; if both cards are the same, they press a button-box that is on the table in front of them as quickly as possible. For each of the 12 rounds, the following data were collected: the pictures shown on the cards (Index of card A, Index of card B), the number of times the participant clicked the 'snap' button, and the time it took to first click the 'snap' button.

In this exploratory factor analysis, we used the mean time to correctly identify matches. This field is the mean duration to first press of snap-button summed over rounds in which both cards matched. It gives a crude measure of the raw processing + reaction speed of a participant.

The following data points were excluded when forming the average:

• Rounds 0-4 were regarded as "training”

• Times under 50ms must be due to anticipation rather than reaction

• Times over 2000ms were ignored as the cards had disappeared by then

Values were rounded to the nearest whole number.

***Number of puzzles correctly solved (Field ID 6373) Category Matrix Pattern Completion***

In this category participants were presented with a series of matrix pattern blocks with an element missing and asked to select the element that best completed the pattern from a range of displayed choices. There are four questions namely number of puzzles correctly solved, number of puzzles viewed, item selected for each puzzle and duration spent answering each puzzle.

For this factor analysis, we have used the number of puzzles correctly solved. This is the number of puzzles for which the participant gave the correct solution. It should be used in conjunction with Field 6374 which describes the number of puzzles attempted. The range is 0-15.

***Number of symbol digit matches made correctly (field ID 23324) Category Symbol Digit Substitution***

In this category participants were presented with one grid linking symbols to single-digit integers and a second grid containing only the symbols. They were then asked to indicate the numbers attached to each of the symbols in the second grid using the first one as a key.

For the factor analysis, we have used the number of symbol digit matches made correctly. This is the number of symbols correctly matched to digits by the participant.

Note that it would be possible to get a high score here simply by repeated entering "1" very quickly as it would be correct 1/8 of the time and a large number of attempts could be made. For this reason, the score must be interpreted alongside Field 6774 which shows the total number of attempts

***Number of puzzles correct (Field ID 21004) Category Tower Rearranging***

In this category participants were presented with an illustration of three pegs (towers) on which three differently colored hoops had been placed. They were then asked to indicate how many moves it would take to re-arrange the hoops into another specific position.

For this factor analysis, we have used the number of puzzles correct. This is the score from the participant answers in Field 6312 [The values the participant entered when shown each puzzle].

***Duration to complete alphanumeric path (trail #2) Field ID 6350) Category Trail Making***

In this category, participant was presented with sets of digits/letters in circles scattered around the screen and asked to click on them sequentially according to a specific algorithm.

In this analysis, we used the duration to complete alphanumeric path (trail #2), and zero represents “Trail not completed”

***Number of word pairs correctly associated (Field ID 20197) Category Paired Associate learning***

In the paired associate learning test the participants were shown 12 pairs of words (for 30 seconds in total) then, after an interval (in which they did a different test), presented with the first word of 10 of these pairs and asked to select the matching second word from a choice of 4 alternatives. The words were presented in the order: huge, happy, tattered, old, long, red, sulking, pretty, tiny, and new.

For the factor analysis, we used the number of word pairs correctly associated out of ten attempts.

# Supplementary Results

**Supplementary Figure 1.** Flowchart of UKB participants included in the combined analyses


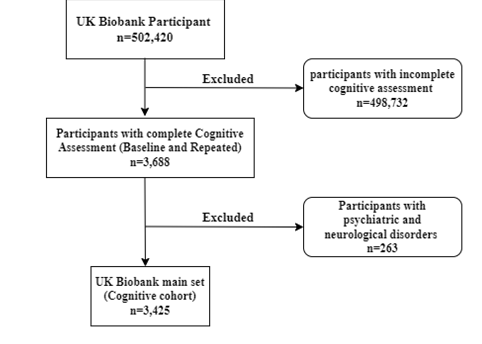


**Supplementary Table 2.** Clinical diseases excluded from the analysis

| Descriptions | n |
| --- | --- |
| Dementia | 0 |
| Schizophrenia | 2 |
| Manic episode | 1 |
| Bipolar disorder | 7 |
| Major depressive disorder | 136 |
| Major depressive disorder, recurrent | 4 |
| Cyclothymic disorder | 0 |
| Unspecified mood [affective] disorder | 0 |
| Agoraphobia and other fear | 7 |
| Panic disorder and other mixed disorder | 82 |
| Mixed obsessional thoughts and acts | 2 |
| Post-traumatic stress disorder (PTSD) | 7 |
| Dissociative amnesia | 0 |
| Somatization disorder | 2 |
| Depersonalization-derealization syndrome | 0 |
| Eating disorder | 1 |
| Primary insomnia | 0 |
| Hypoactive sexual desire disorder | 3 |
| Puerperal psychosis | 0 |
| Psychological and behavioral factors associated with disorders classified elsewhere | 0 |
| Abuse of non-psychoactive substances | 0 |
| Personality disorder | 3 |
| Pathological gambling | 0 |
| Gender identity disorders | 0 |
| Paraphilias | 0 |
| Other sexual disorders | 0 |
| Intellectual disability | 0 |
| Specific developmental disorders of speech and language | 2 |
| Attention-deficit hyperactivity disorders | 2 |
| Parkinson's disease | 12 |
| Alzheimer's disease | 2 |
| Multiple sclerosis | 12 |
| stroke | 4 |
| Alcohol abuse | 18 |
| Alcohol dependence | 13 |
| Alcohol use unspecified with intoxication | 0 |
| Opioid abuse, dependence, and related disorders | 0 |
| Cannabis abuse, dependence, and related disorders | 0 |
| Sedative, hypnotic, or anxiolytic abuse | 0 |
| Cocaine abuse, dependence, and related disorders | 0 |
| Other stimulant abuse, dependence, and related disorders | 0 |
| Hallucinogen abuse, dependence, and related disorders | 0 |
| Nicotine dependence, unspecified, uncomplicated | 0 |
| Inhalant use and related disorders | 0 |
| Other psychoactive substance dependence | 0 |

## Supplementary Table 3. Pearson’s Correlation Matrix for The Nine Cognitive Tests in The UK Biobank

|  | FI | NM | PM | RT | MPC | SDS | TR | TM | PAL |
| --- | --- | --- | --- | --- | --- | --- | --- | --- | --- |
| FI | 1.00 |  |  |  |  |  |  |  |  |
| NM | 0.35 | 1.00 |  |  |  |  |  |  |  |
| PM | -0.13 | -0.11 | 1.00 |  |  |  |  |  |  |
| RT | -0.10 | -0.05 | 0.13 | 1.00 |  |  |  |  |  |
| MPC | 0.38 | 0.23 | -0.19 | -0.17 | 1.00 |  |  |  |  |
| SDS | 0.25 | 0.15 | -0.19 | -0.24 | 0.36 | 1.00 |  |  |  |
| TR | 0.31 | 0.21 | -0.20 | -0.20 | 0.35 | 0.35 | 1.00 |  |  |
| TM | -0.28 | -0.22 | 0.14 | 0.13 | -0.28 | -0.31 | -0.25 | 1.00 |  |
| PAL | 0.31 | 0.21 | -0.17 | -0.11 | 0.28 | 0.25 | 0.25 | -0.19 | 1.00 |

**Abbreviations**: FI=Fluid intelligence; NM=Numeric memory; PM=Pairs matching; RT=Reaction time; MPC=Matrix pattern completion; SDS=Symbol digit substitution; TR=Tower rearranging; TM=Trail making; PAL=Paired associate learning

**Supplementary Figure 2.** Graphical presentation of the relationships of the nine cognitive tests in the UK Biobank. Scatter plots are below diagonal. An ellipse around the mean with the axis length reflecting one standard deviation of the x and y variables is also drawn. The x-axis in each scatter plot represents the column variable, the y-axis shows the row variable. Histograms of each variable are presented on the diagonal. The upper part of the diagonal is the Pearson correlation of the nine cognitive tests in the UKB.


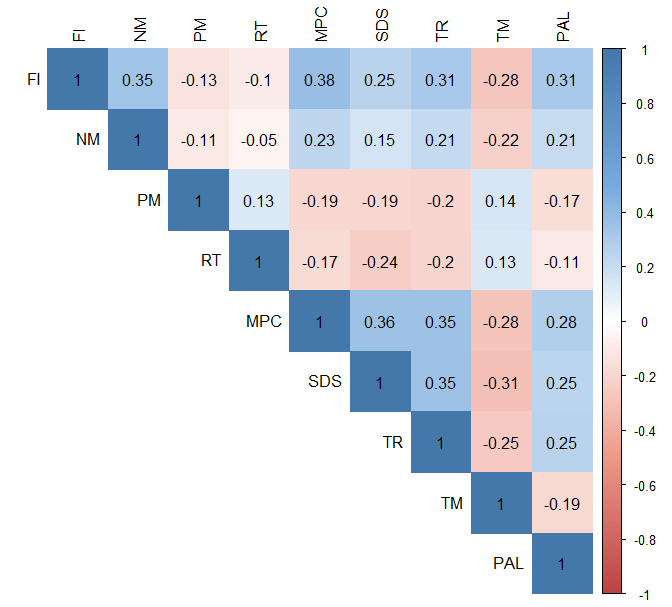


**Abbreviations**: FI=Fluid intelligence; NM=Numeric memory; PM=Pairs matching; RT=Reaction time; MPC=Matrix pattern completion; SDS=Symbol digit substitution; TR=Tower rearranging; TM=Trail making; PAL=Paired associate learning

**Exploratory Factor Analysis [Using nine cognitive tests]**

This factor analysis is conducted using the *psych* R package. The *factanal()* function in the package produces a maximum likelihood factor analysis. Using describe function in the *psych* page provides the basic descriptive statistic arranged in a data frame format and scatter plot matrices (SPLOMS) for the nine cognitive tests in the UK Biobank

**Supplementary Figure 3.** Graphical presentation of the relationships of the nine cognitive tests in the UK Biobank***.*** Scatter plots are below diagonal. An ellipse around the mean with the axis length reflecting one standard deviation of the x and y variables is also drawn. The x-axis in each scatter plot represents the column variable, the y-axis shows the row variable. Histograms of each variable are presented on the diagonal. The upper part of the diagonal is the Pearson correlation of the nine cognitive tests in the UKB.


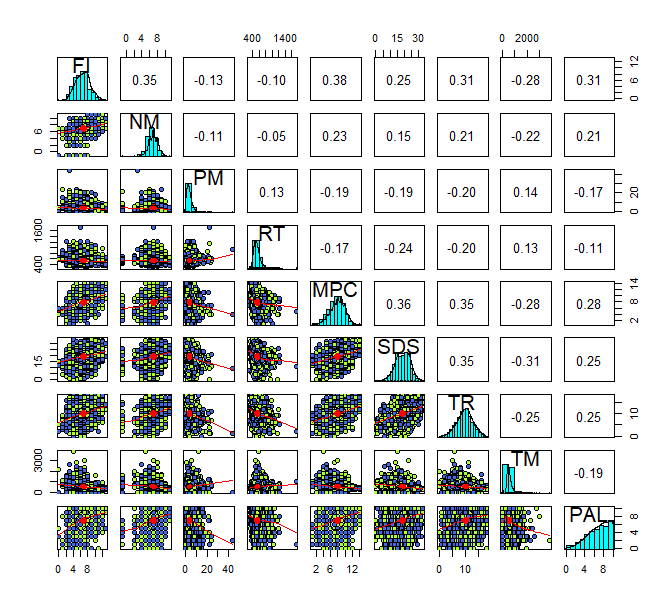


**Abbreviations**: FI=Fluid intelligence; NM=Numeric memory; PM=Pairs matching; RT=Reaction time; MPC=Matrix pattern completion; SDS=Symbol digit substitution; TR=Tower rearranging; TM=Trail making; PAL=Paired associate learning

**Supplementary Table 4.** Eigenvalues for the nine cognitive tests in the UK Biobank for the scree analysis.

| No | Factors | Eigenvalue |
| --- | --- | --- |
| 1 | Fluid intelligence | 2.863 |
| 2 | Numeric memory | 1.076 |
| 3 | Pairs matching | 0.887 |
| 4 | Reaction time | 0.811 |
| 5 | Matrix pattern completion | 0.792 |
| 6 | Symbol digit solution | 0.722 |
| 7 | Tower rearranging | 0.654 |
| 8 | Trail making | 0.625 |
| 9 | Paired associate learning | 0.565 |

The above table of eigenvalue suggests the number of factors to extract is two. In addition, the parallel analysis suggests 2 factors to extract. Furthermore, we extend the analysis using the VSS technique was to determine the number of factors to extract. VSS applies goodness of fit test to determine the optimal number of factors to extract. It can be thought of as a quasi-confirmatory model, in that it fits the very simple structure (all except the biggest c loadings per item are set to zero where c is the level of complexity of the item) of a factor pattern matrix to the original correlation matrix. For items where the model is usually of complexity one, this is equivalent to making all except the largest loading for each item 0. The VSS function compares the fit of a number of factors analyses with the loading matrix “simplified” by deleting all except the C greatest loading per item, where C is a measure of factor complexity. Included in VSS is the MAP criterion (Minimum Absolute Partial correlation).

Using the VSS criterion for the nine cognitive tests in the UK Biobank suggests that four factors are optimal that explain the maximum amount of variability in the data. However, the Velicer MAP criterion suggests that a one-factor solution is optimal.

**Supplementary Figure 4.** The very Simple Structure criterion for the number of factors compares solutions for various levels of item complexity and various numbers of factors.

The plot shows the number of factors on the x-axis and the VSS (Complexity) fit along the y-axis with lines and numbers in the Cartesian plane representing the different factor models. For the nine cognitive tests in the UK Biobank, the complexity 1 and 2 solutions achieve their maxima at one and four factors respectively. This contrasts with parallel analysis which suggests two factors and the MAP criterion which suggests one factor.


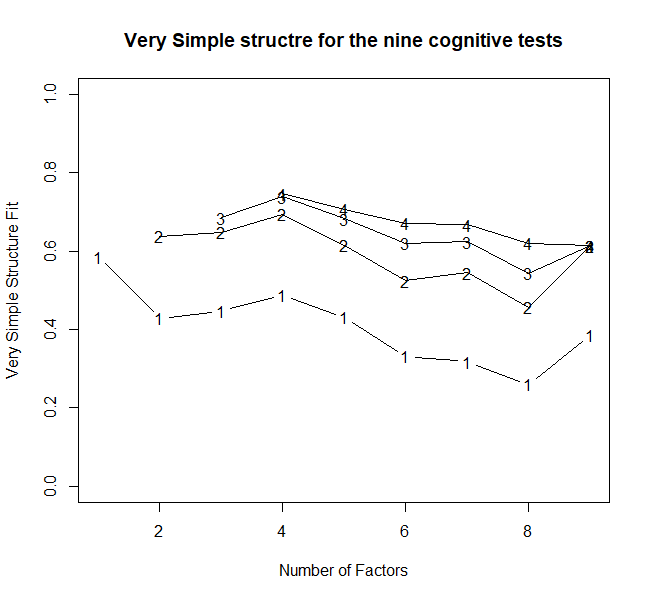


**Supplementary Table 5.** A simple structure analysis for the nine cognitive function tests in the UK Biobank

| Vss complexity 1 achieves a maximum of 0.59 with 1 factor | | | | | | | | | | | | | | | |
| --- | --- | --- | --- | --- | --- | --- | --- | --- | --- | --- | --- | --- | --- | --- | --- |
| Vss complexity 2 achieves a maximum of 0.69 with 4 factors | | | | | | | | | | | | | | | |
| The Velicer MAP achieves a minimum of 0.02 with 1 factor | | | | | | | | | | | | | | | |
| BIC achieves a minimum of -116.27 with 2 factors | | | | | | | | | | | | | | | |
| Sample size adjusted BIC achieves a minimum of -55.89 with 2 factors | | | | | | | | | | | | | | | |
| Statistics by number of factors | | | | | | | | | | | | | | | |
| **Vss1** | **Vss2** | **map** | **dof** | **chisq** | **prob** | **sqresid** | **Fit** | **RMSEA** | **BIC** | **SABIC** | **complex** | **eChisq** | **SRMR** | **eCRMS** | **eBIC** |
| 0.59 | 0.00 | 0.019 | 27 | 3.0e+02 | 2.4e-48 | 5.4 | 0.59 | 0.0546 | 82.6 | 168.4 | 1.0 | 3.8e+02 | 3.9e-02 | 0.0456 | 164.4 |
| 0.43 | 0.64 | 0.039 | 19 | 3.8e+01 | 5.3e-03 | 4.7 | 0.64 | 0.0173 | -116.3 | -55.9 | 1.5 | 4.3e+01 | 1.3e-02 | 0.0181 | -111.8 |
| 0.45 | 0.65 | 0.076 | 12 | 1.5e+01 | 2.3e-01 | 4.1 | 0.69 | 0.0087 | -82.3 | -44.4 | 1.5 | 1.7e+01 | 8.4e-03 | 0.0145 | -80.5 |
| 0.49 | 0.69 | 0.131 | 6 | 3.8e+00 | 7.0e-01 | 3.3 | 0.75 | 0.0000 | -45.0 | -25.9 | 1.5 | 3.9e+00 | 4.0e-03 | 0.0097 | -45.0 |
| 0.43 | 0.62 | 0.203 | 1 | 1.3e+00 | 2.5e-01 | 3.8 | 0.71 | 0.0093 | -6.8 | -3.7 | 1.8 | 1.4e+00 | 2.4e-03 | 0.0144 | -6.7 |
| 0.33 | 0.53 | 0.308 | -3 | 1.6e+06 | NA | 4.0 | 0.69 | NA | NA | NA | 2.3 | 1.6e-06 | 2.5e-06 | NA | NA |
| 0.32 | 0.55 | 0.440 | -6 | 4.1e-08 | NA | 4.1 | 0.68 | NA | NA | NA | 2.4 | 4.6e-08 | 4.3e-07 | NA | NA |
| 0.26 | 0.46 | 1.000 | -8 | 6.1e-12 | NA | 4.0 | 0.69 | NA | NA | NA | 2.9 | 6.5e-19 | 1.6e-12 | NA | NA |
| 0.39 | 0.61 | NA | -9 | 9.1e+01 | NA | 5.1 | 0.61 | NA | NA | NA | 1.6 | 1.2e+02 | 2.2e-02 | NA | NA |

The optimal number of factors to extract is not the same in all methods applied. For example, the parallel analysis suggests two factors, VSS suggests four factors with a complexity of 2 and the Velicer MAP suggests 1 factor is optimum. We applied a different grid search of optimum numbers that range from two to four-factor solutions are included. Finally, the three-factor solution was the optimum factor with meaningful interpretations.

**Supplementary Table 6.** Three-factors solution using the nine cognitive function tests in the UK Biobank

| **Uniqueness**: | | | | | | | | |
| --- | --- | --- | --- | --- | --- | --- | --- | --- |
| FI | NM | PM | RT | MPC | SDS | TR | TM | PAL |
| 0.476 | 0.770 | 0.887 | 0.854 | 0.623 | 0.598 | 0.657 | 0.005 | 0.776 |
| **Loadings**: |  |  |  |  |  |  |  |  |
|  |  | Factor 1 | Factor 2 | Factor 3 |  |  |  |  |
| Fluid intelligence | |  | 0.746 |  |  |  |  |  |
| Numeric memory | |  | 0.492 |  |  |  |  |  |
| Pairs matching | | -0.343 |  |  |  |  |  |  |
| Reaction time | | -0.469 | 0.162 |  |  |  |  |  |
| Matrix pattern completion | | 0.412 | 0.265 |  |  |  |  |  |
| Symbol digit substitution | | 0.663 |  |  |  |  |  |  |
| Tower rearranging | | 0.500 | 0.132 |  |  |  |  |  |
| Trail making | |  |  | 0.973 |  |  |  |  |
| Paired associate learning | | 0.229 | 0.308 |  |  |  |  |  |
|  | | | | | | | | |
|  |  | Factor 1 | Factor 2 | Factor 3 |  |  |  |  |
| SS loadings | | 1.256 | 1.014 | 0.953 |  |  |  |  |
| Proportion var | | 0.140 | 0.113 | 0.106 |  |  |  |  |
| Cumulative var | | 0.140 | 0.252 | 0.358 |  |  |  |  |
|  |  |  |  |  |  |  |  |  |
| Factor Correlations: | | |  |  |  |  |  |  |
|  | Factor 1 | Factor 2 | Factor 3 |  |  |  |  |  |
| Factor 1 | 1.000 | -0.370 | -0.394 |  |  |  |  |  |
| Factor 2 | -0.370 | 1.000 | 0.643 |  |  |  |  |  |
| Factor 3 | -0.394 | 0.643 | 1.000 |  |  |  |  |  |
| Test of the hypothesis that 3 factors are sufficient: | | | | | | | | |
| The chi-square statistics is 15.14 on 12 degrees of freedom | | | | | | | | |
| The p-value is 0.234 | | | | | | | | |

**Abbreviations**: FI=Fluid intelligence; NM=Numeric memory; PM=Pairs matching; RT=Reaction time; MPC=Matrix pattern completion; SDS=Symbol digit substitution; TR=Tower rearranging; TM=Trail making; PAL=Paired associate learning.

**Supplementary Table 7.** Loading score for three-factors solution model using nine cognitive function tests

| **Loadings** | | | |
| --- | --- | --- | --- |
|  | Factor 1 | Factor 2 | Factor 3 |
| Symbol digit substitution | 0.663 |  |  |
| Fluid intelligence |  | 0.746 |  |
| Trail making |  |  | 0.973 |
| Numeric memory |  | 0.492 |  |
| Pairs matching | -0.343 |  |  |
| Reaction time | -0.469 |  |  |
| Matrix pattern completion | 0.412 | 0.265 |  |
| Tower rearranging | 0.500 |  |  |
| Paired associated learning | 0.229 | 0.308 |  |
|  |  |  |  |
|  | Factor 1 | Factor 2 | Factor 3 |
| SS loadings | 1.256 | 1.014 | 0.953 |
| Proportion var | 0.140 | 0.113 | 0.106 |
| Cumulative var | 0.140 | 0.252 | 0.358 |
| **Correlation** |  |  |  |
|  | Factor 1 | Factor 2 | Factor 3 |
| Factor 1 | 1.00 | -0.34 | 0.20 |
| Factor 2 | -0.34 | 1.00 | 0.20 |
| Factor 3 | 0.20 | 0.20 | 1.00 |
|  |  |  |  |

Interpretation: Uniqueness is the variance unexplained by the linear combination of the factors and unique to that specific factor. High uniqueness indicates that the factors do not account well for its variance. Loadings are the contribution of each original variable to the factor. Variables with a high loading are well explained by the factor. For some variables, there is no loading as R does not print loading less than 0.1. The value beneath the loadings shows the proportion of variance explained by each factor. The row Cumulative Var [ranges from 0 to 1] gives the cumulative proportion of variance explained. The row Proportion Var gives the proportion of variance explained by each factor. The row SS loadings give the sum of squared loadings. A factor is worth keeping if the SS loading is greater than 1 (Kaiser’s rule). The last section is the hypothesis test result. Conventionally, we do not reject the null hypothesis [H0: the number of factors in the model is sufficient, in our case 3 factors] if the P-value is greater than 0.05. The high P-value [0.234] in our 3-factor solution leads us to not reject the null hypothesis and indicates that we fitted an appropriate model using the maximum likelihood estimation to capture the full dimensionality of the dataset. The factor pattern is cleaned using the cutoff value (0.2) to hide small loadings, to reduce the visual clutter in the factor pattern. In addition, the loadings are sorted.

**Exploratory Structural Equation Modelling (ESEM)**

**Supplementary Table 8.** The two-factor solution loadings and proposed model for the ESEM

|  | Factor 1 | Factor 2 | Factor 3 | Factor 1 | Factor 2 | Factor 3 |
| --- | --- | --- | --- | --- | --- | --- |
| Fluid intelligence |  | 0.746 |  |  | 0.746 |  |
| Numeric memory |  | 0.492 |  |  | 0.492 |  |
| Pairs matching | -0.343 |  |  |  |  | 0.300 |
| Reaction time | -0.469 | 0.162 |  |  |  | 0.300 |
| Matrix pattern completion | 0.412 | 0.265 |  | 0.412 | 0.300 |  |
| Symbol digit substitution | 0.663 |  |  | 0.663 |  |  |
| Tower rearranging | 0.500 | 0.132 |  | 0.500 |  |  |
| Trail making |  |  | 0.973 |  |  | 0.700 |
| Paired associate learning | 0.229 | 0.308 |  |  | 0.308 |  |

This is an indicator with high loading in one factor and low loading in all the others. In this analysis the anchor for F1= [”X5” +”X6” +”X7], F2= [”X1” +”X2” +”X5” +”X9”] and F3= [”X3” +”X4” +”X8”]. These values are highlighted in the above table. In setting the model, we set the cross-loadings values from the three-factor solution. The values for the marking are displayed below (latent variable definitions)

F1 =~ (0.412) * x5 + (0.663) *x6 + (0.500) *x7

F2 =~ (0.746) *x1 + (0.492) *x2 + (0.300) *x5 + (0.308) *x9

F3 =~ (0.300) *x3 + (0.300) *x4 + (0.700) *x8

Note: X5 is assigned with different loadings [same test loads two factors].

Where x1= fluid intelligence; x2=numeric memory; x3=pairs matching; x4=reaction time; x5=matrix pattern completion; x6=symbol digit substitution; x7=tower rearranging; x8=trail making; x9=paired associate learning. The model fit used Gaussian distribution that best describes a given empirical sample.”

**Supplementary Table 9.** Two-factors solution analysis of cognitive function measurement in the UK Biobank using oblimin rotation

| Call factanal(x = items, factors = 2, n.obs = 3425, rotation = "oblimin") | | | | | | | | | |
| --- | --- | --- | --- | --- | --- | --- | --- | --- | --- |
| Uniqueness: | | | | | | | | | |
| FI | NM | PM | RT | MPC | SDS | TR | TM | PAL | |
| 0.487 | 0.763 | 0.891 | 0.858 | 0.627 | 0.573 | 0.667 | 0.779 | 0.782 | |
| Loadings: |  |  |  |  |  |  |  |  | |
|  |  | Factor 1 | Factor 2 |  |  |  |  |  | |
| Fluid intelligence | |  | 0.71 |  |  |  |  |  | |
| Numeric memory | |  | 0.50 |  |  |  |  |  | |
| Pairs matching | | -0.32 |  |  |  |  |  |  | |
| Reaction time | | -0.44 |  |  |  |  |  |  | |
| Matrix pattern completion | | 0.40 | 0.28 |  |  |  |  |  | |
| Symbol digit substitution | | 0.68 |  |  |  |  |  |  | |
| Trail making | | -0.32 |  |  |  |  |  |  | |
| Tower rearranging | | 0.47 |  |  |  |  |  |  | |
| Paired associate learning | | 0.21 | 0.31 |  |  |  |  |  | |
|  | | | | | | | | | |
|  | | Factor 1 | Factor 2 |  |  |  |  |  | |
| SS loadings | | 1.29 | 1.02 |  |  |  |  |  | |
| Proportion var | | 0.14 | 0.11 |  |  |  |  |  | |
| Cumulative var | | 0.14 | 0.26 |  |  |  |  |  | |
|  |  |  |  |  |  |  |  |  | |
| Factor Correlations: | | |  |  |  |  |  |  |  |
|  | Factor 1 | Factor 2 |  |  |  |  |  |  | |
| Factor 1 | 1.00 | -0.31 |  |  |  |  |  |  | |
| Factor 2 | -0.31 | 1.00 |  |  |  |  |  |  | |
| Test of the hypothesis that 2 factors are sufficient: | | | | | | | | | |
| The chi-square statistics is 38.37 on 19 degrees of freedom | | | | | | | | | |
| The p-value is 0.00532 | | | | | | | | | |

**Abbreviations**: FI=Fluid intelligence; NM=Numeric memory; PM=Pairs matching; RT=Reaction time; MPC=Matrix pattern completion; SDS=Symbol digit substitution; TR=Tower rearranging; TM=Trail making; PAL=Paired associate learning.

**Supplementary Table 10.** Three-factors solution analysis of cognitive function measurement in the UK Biobank using oblimin rotation

| Call factanal(x = items, factors = 3, n.obs = 3425, rotation = "oblimin") | | | | | | | | |
| --- | --- | --- | --- | --- | --- | --- | --- | --- |
| **Uniqueness**: | | | | | | | | |
| FI | NM | PM | RT | MPC | SDS | TR | TM | PAL |
| 0.476 | 0.770 | 0.887 | 0.854 | 0.623 | 0.598 | 0.657 | 0.005 | 0.776 |
| **Loadings**: | | | | | | | | |
|  | | Factor 1 | Factor 2 | Factor 3 |  |  |  |  |
| Fluid intelligence | |  |  | 0.71 |  |  |  |  |
| Numeric memory | |  |  | 0.47 |  |  |  |  |
| Pairs matching | | -0.33 |  |  |  |  |  |  |
| Reaction time | | -0.45 |  |  |  |  |  |  |
| Matrix pattern completion | | 0.41 |  | 0.27 |  |  |  |  |
| Symbol digit substitution | | 0.63 |  |  |  |  |  |  |
| Trail making | |  | 0.99 |  |  |  |  |  |
| Tower rearranging | | 0.50 |  |  |  |  |  |  |
| Paired associate learning | | 0.24 |  | 0.30 |  |  |  |  |
|  | | | | | | | | |
|  | | Factor 1 | Factor 2 | Factor 3 |  |  |  |  |
| SS loadings | | 1.183 | 1.001 | 0.934 |  |  |  |  |
| Proportion var | | 0.131 | 0.111 | 0.104 |  |  |  |  |
| Cumulative var | | 0.131 | 0.243 | 0.346 |  |  |  |  |
|  | | | | | | | | |
| Factor Correlations: | | |  |  |  |  |  |  |
|  | Factor 1 | Factor 2 | Factor 3 |  |  |  |  |  |
| Factor 1 | 1.00 | 0.27 | -0.22 |  |  |  |  |  |
| Factor 2 | 0.27 | 1.00 | 0.20 |  |  |  |  |  |
| Factor 3 | -0.22 | 0.20 | 1.00 |  |  |  |  |  |
| Test of the hypothesis that 3 factors are sufficient: | | | | | | | | |
| The chi-square statistics is 15.14 on 12 degrees of freedom | | | | | | | | |
| The p-value is 0.234 | | | | | | | | |

**Abbreviations**: FI=Fluid intelligence; NM=Numeric memory; PM=Pairs matching; RT=Reaction time; MPC=Matrix pattern completion; SDS=Symbol digit substitution; TR=Tower rearranging; TM=Trail making; PAL=Paired associate learning.

**Supplementary Table 11.** Three-factors solution with eight cognitive tests [excluding trail making] in the UK Biobank using oblimin rotation

| Call factanal(x = items, factors = 3, n.obs = 3425, rotation = "oblimin") | | | | | | | | |
| --- | --- | --- | --- | --- | --- | --- | --- | --- |
| **Uniqueness**: | | | | | | | | |
| FI | NM | PM | RT | MPC | SDS | TR | PAL |  |
| 0.478 | 0.771 | 0.005 | 0.857 | 0.624 | 0.582 | 0.664 | 0.778 |  |
| **Loadings**: | | | | | | | | |
|  | | Factor 1 | Factor 2 | Factor 3 |  |  |  |  |
| Fluid intelligence | |  |  | 0.72 |  |  |  |  |
| Numeric memory | |  |  | 0.50 |  |  |  |  |
| Pairs matching | |  | 0.99 |  |  |  |  |  |
| Reaction time | | -0.43 |  |  |  |  |  |  |
| Matrix pattern completion | | 0.40 |  | 0.29 |  |  |  |  |
| Symbol digit substitution | | 0.67 |  |  |  |  |  |  |
| Tower rearranging | | 0.45 |  |  |  |  |  |  |
| Paired associate learning | |  |  | 0.32 |  |  |  |  |
|  | | | | | | | | |
|  | | Factor 1 | Factor 2 | Factor 3 |  |  |  |  |
| SS loadings | | 1.030 | 1.002 | 0.989 |  |  |  |  |
| Proportion var | | 0.129 | 0.125 | 0.124 |  |  |  |  |
| Cumulative var | | 0.129 | 0.254 | 0.378 |  |  |  |  |
|  | | | | | | | | |
| Factor Correlations: | | |  |  |  |  |  |  |
|  | Factor 1 | Factor 2 | Factor 3 |  |  |  |  |  |
| Factor 1 | 1.00 | 0.21 | -0.27 |  |  |  |  |  |
| Factor 2 | 0.21 | 1.00 | 0.09 |  |  |  |  |  |
| Factor 3 | -0.27 | 0.09 | 1.00 |  |  |  |  |  |
| Test of the hypothesis that 3 factors are sufficient: | | | | | | | | |
| The chi-square statistics is 3.62 on 7 degrees of freedom | | | | | | | | |
| The p-value is 0.822 | | | | | | | | |

**Abbreviations**: FI=Fluid intelligence; NM=Numeric memory; PM=Pairs matching; RT=Reaction time; MPC=Matrix pattern completion; SDS=Symbol digit substitution; TR=Tower rearranging; TM=Trail making; PAL=Paired associate learning.
